# An efficient extraction device for microplastics in marine sediments and its applications†

**DOI:** 10.1039/d4ra04347b

**Published:** 2024-11-08

**Authors:** Wang Jiahan, Liu Xiaowei, Yang Feng, Yang Xiujiu, Jiao Wenguang, Tang Kai, Wang Jinli, Chen Yan

**Affiliations:** a Haikou Marine Geological Survey Center, China Geological Survey Haikou 571127 China 450733437@qq.com wjhazxy@qq.com

## Abstract

Microplastics, defined as small pieces of plastic with a size less than 5 millimeters, constitute a significant sink for microplastics in marine sediments. Given the potential harm to nature and human beings, accurate detection of microplastics in marine sediments is of the utmost importance. The separation of microplastics from marine sediments represents a pivotal step in the quantitative detection of microplastics. This paper presents a high-efficiency extraction device for microplastics in marine sediments, with the objective of enhancing the effectiveness and efficiency of microplastics extraction. The device employs an air pump to thoroughly mix the samples and incorporates metal perforated plate fillers to achieve efficient sedimentation, thus facilitating the separation of microplastics from the surrounding marine sediments. Subsequently, the separated microplastics are passed through a series of pore sizes of stainless steel screens and glass fibre filters *via* suction filtration, allowing for the collection of microplastics of varying particle sizes for subsequent identification. Following a series of method trials, the optimal extraction conditions for this device were identified. The results demonstrated its excellent extraction effectiveness and high efficiency. To verify the feasibility of this device, it was used to investigate the microplastics in the sediments of Dongzhai Harbor, Hainan. The abundance, particle size distribution, shape, and composition of microplastics in the sediments of this area were obtained, which not only validated the practicality of this microplastic extraction device but also provided significant insights for ecological and environmental protection in the region.

## Abstract 2

Microplastics, defined as small pieces of plastic with a size less than 5 millimeters, constitute a significant sink for microplastics in marine sediments.

## Graphical abstract

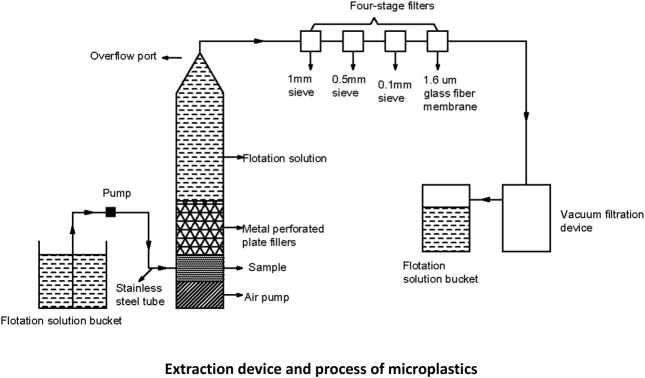

## 1 Introduction

Due to their excellent flexibility, low cost, diverse functionalities, durability, and lightweight properties, plastics have been extensively produced and consumed worldwide. Currently, the global annual production of plastics exceeds 320 million tons (Mt), with disposable plastics accounting for more than 40% of the total.^1^ Among all plastic waste, microplastics (MPs) have garnered the most attention. Microplastics are defined as small pieces of plastic with a size less than 5 millimetres that ultimately enter the natural environment.^2^ Marine sediments serve as a significant sink^3^ for microplastics, and even in remote areas far from human activity centres,^4^ such as the sediments of the Pacific,^5^ Atlantic Oceans^6^ and Antarctica,^7^ microplastics are frequently detected. The potential hazards of microplastics have attracted considerable attention, with the majority of research focusing on the following aspects: firstly, microplastics may have a significant impact on the element cycling in sediments, both directly and indirectly. As microplastics are rich in carbon, the increased carbon storage can directly affect the carbon cycling process, potentially altering carbon sequestration and emissions in sediments.^1^ Furthermore, under the influence of solar radiation and biodegradation, low-molecular-weight polymers within microplastics may be released. These released polymers are perceived as available carbon sources by microorganisms,^8,9^ thereby affecting the carbon, nitrogen, phosphorus, and sulfur cycling of microbial communities, disturbing the ecological balance.^1^ Secondly, microplastics may directly harm organisms. The ingestion of microplastics by organisms can result in a number of adverse effects, including fatigue, decreased appetite, blocked metabolic pathways, and changes in feeding behaviour. These effects pose a significant risk to human health.^10,11^

In conclusion, the accurate detection of microplastics in marine sediments is of paramount importance. The quantitative detection of microplastics involves three main steps: sampling, sample preparation, and analysis.^12,13^ Among these, sample preparation is a critical and time-consuming step, with the core objective being to separate microplastics from marine sediments. The majority of studies related to this process employ density separation techniques,^14–16^ which operate on the principle that minerals have a density range of 2.5 g cm^−3^ to 2.8 g cm^−3^, while microplastics have a density range of 0.05 g cm^−3^ to 2.3 g cm^−3^.^17^ When samples are placed in a flotation liquid, the combined effects of gravity and buoyancy result in the separation of microplastics from sediments. This is achieved by the rise of microplastics and the sinking of sediments. Commonly chosen flotation liquids include saturated solutions of NaCl (1.2 g cm^−3^), ZnBr_2_ (1.71 g cm^−3^), ZnCl_2_ (1.7 g cm^−3^), and NaI (1.8 g cm^−3^).^18–22^ The traditional density separation method involves the addition of a flotation liquid to the sediment, stirring, and then allowing the mixture to settle before the microplastics are floated out. However, this method has several apparent drawbacks. For instance, it can often result in incomplete extraction, requiring high levels of expertise from operators. Moreover, the entire process is cumbersome, with the need for long settling periods of the solution, which ultimately reduces extraction efficiency. For instance, Fries *et al.*^23^ employed sodium chloride as the flotation liquid and utilised a funnel to separate microplastics from beach sand. Each sample necessitated repeated operations on two occasions, and due to the simplicity of the separation method, the separation efficiency was relatively low (over 80%). In another instance, Wazne *et al.*^24^ implemented minor enhancements to the conventional sediment microplastic separation technique by utilising a commonly available glass separating funnel for the extraction of microplastics. While this method effectively alleviated the frequent clogging issues encountered in density separation devices, the need for a 24 hours settling period after the addition of the flotation liquid and sediment still resulted in low extraction efficiency. In recent years, researchers have attempted to utilise automatic flotation devices as a substitute for manual operations to enhance the separation efficiency of microplastics in sediments. For instance, Coppock *et al.*^25^ designed a cylindrical separation device utilising zinc chloride as the flotation liquid, which is portable and boasts high separation efficiency. However, the device suffers from inadequate mixing between the flotation liquid and the samples, and the microplastics tend to adhere to the cylinder walls, affecting their recovery. Furthermore, the flotation liquid cannot be recycled. Han *et al.*^26^ created an air mixing and flotation device that employs sodium chloride and sodium iodide as the flotation liquid for separating microplastics from soil or sediment. The use of aeration enables thorough mixing of the flotation liquid and samples, resulting in a high separation efficiency of over 90%. However, the procedure is intricate and challenging to operate. Liu *et al.*^27^ designed a continuous automatic separation device that selects sodium bromide as the flotation liquid for isolating microplastics from soil samples. While the used flotation liquid can be recycled through a flotation liquid recovery unit, the lengthy precipitation time required after sample mixing reduces the extraction efficiency. Liu *et al.*^28^ also developed a rapid flotation and automatic graded separation method for microplastics, achieving efficient flotation of microplastics from sediments through micro-bubble aeration and the efficient sedimentation effect of ceramic corrugated fillers. However, the device is reliant on the use of gas cylinders for the supply of air, which is inconvenient and expensive to replace. Furthermore, the separation of microplastics and flotation liquid at the overflow outlet of the device is not entirely thorough.

This paper addresses the aforementioned issues by presenting a highly efficient extraction device for microplastics in marine sediments. The device employs an air pump for sample mixing and metal perforated plate fillers for efficient sedimentation, thereby enabling the separation of microplastics from marine sediments. The separated microplastics are then filtered through different aperture sizes of stainless steel sieves and glass fibre membranes using suction filtration, resulting in microplastics of various particle sizes. It is noteworthy that the flotation liquid can be recycled for reuse. The device offers a comprehensive extraction process and straightforward operation, thereby markedly reducing the extraction time. To validate the feasibility of this device, a survey was conducted using it to investigate the microplastics in the sediments of Dongzhai Harbor, Hainan. The survey provided insights into the abundance, particle size distribution, shape, and composition of microplastics in the sediment of this region. Moreover, this study validates the practicality of the microplastic extraction device designed in this paper and provides crucial reference information for ecological and environmental protection in the area.

## 2 Materials and methods

### 2.1 Materials and instruments

Primary materials and accessories: stainless steel cylinder (Jiangsu Huasheng Company); air pump (Kachuaner Fluid Technology (Shanghai) Company); titanium alloy microporous aerator (Kachuaner Fluid Technology (Shanghai) Company, pore size: 0.45–100 μm, porosity: 35–50%, bubble diameter: 0.1–2 mm); diaphragm pump (Kachuaner Fluid Technology (Shanghai) Company); filtration unit (Haining Delfilter New Material Technology Company); stainless steel detachable filter (Haining Delfilter New Material Technology Company); metal perforated plate packing (Pingxiang Yangrong Chemical Filler Company).

Instruments: JSZ6S Stereoscopic Microscope (Nanjing Jiangnan Yongxin Optics Company); INVENIO Fourier Transform Infrared Spectrometer (Bruker Corporation, Germany).

### 2.2 Principle and design of separation methods

Currently, density separation is a commonly employed method for the separation of microplastics from marine sediments. Based on this principle, some scholars have proposed the use of air flotation for the separation of microplastics from sediments. The principle is to inject a large quantity of bubbles into the sample and flotation liquid, so that the bubbles can adsorb the microplastics in the sample and carry them to the surface of the liquid, thereby achieving the separation purpose.

Furthermore, the conventional density separation methodology employs the density disparity between sediments and microplastics to separate them in a static state. Some scholars have proposed that during the sedimentation phase of microplastics, the utilisation of inclined and tortuous channels can enhance the precipitation area of sediments and microplastics in the flotation liquid,^28^ thereby accelerating the sedimentation of microplastics. The principles outlined above have been applied to the design of the device described in this article. An air pump is connected to a titanium alloy microporous aerator to provide uniform and fine bubbles for the reaction. The fine bubbles adsorb the microplastics in the flotation liquid and, under the action of buoyancy, cause the microplastics to float to the top of the flotation liquid, thus achieving the separation effect.

### 2.3 Device construction

**Fig. 1:**
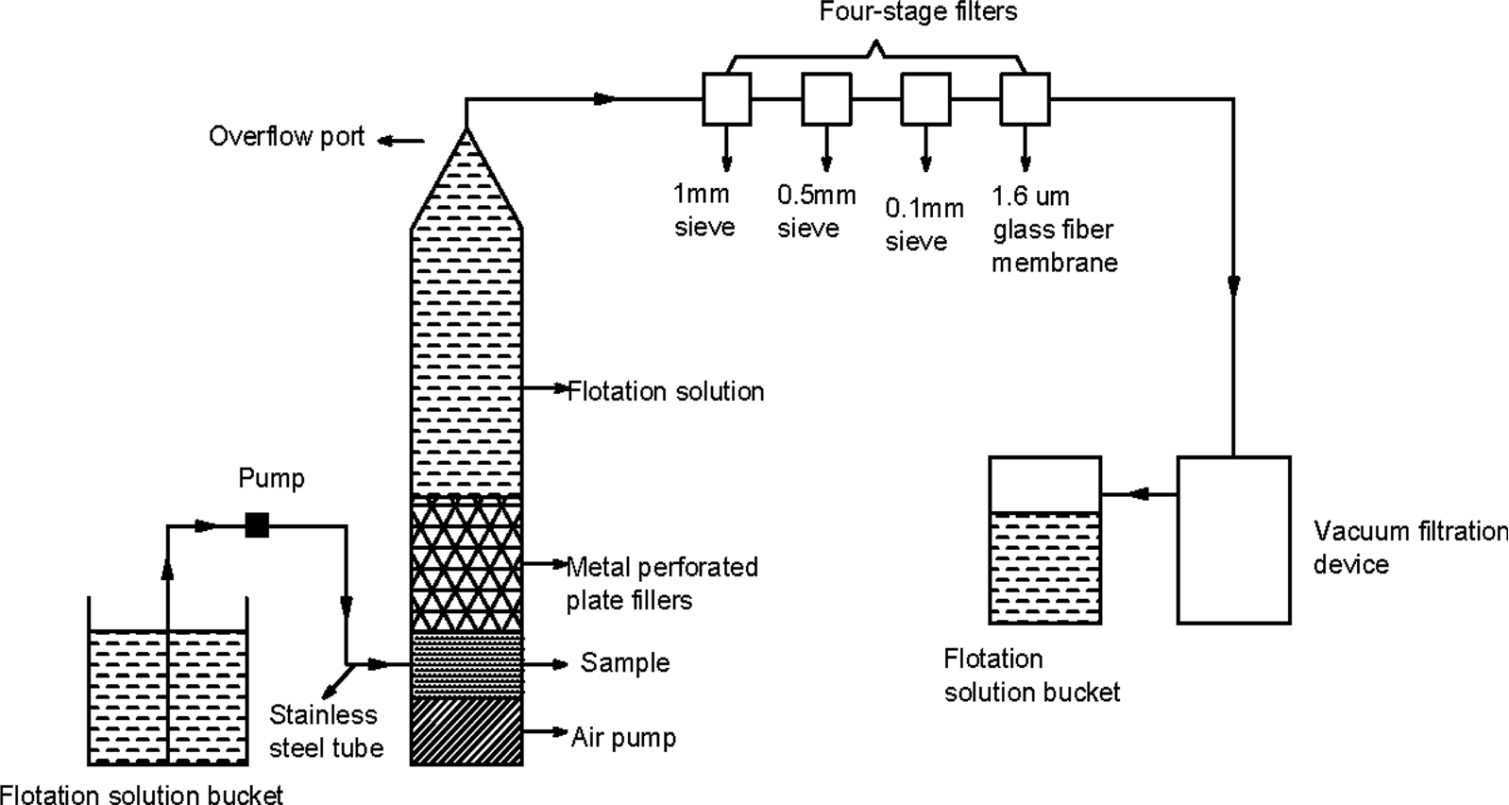
Schematic diagram of microplastic extraction device.

The microplastic separation device described in this article is composed of a flotation liquid tank, a water pump (diaphragm pump), stainless steel tubes, an air pump, a cylindrical stainless steel cylinder, metal perforated plate packing, a conical overflow port, a filter, and a suction filtration unit, as illustrated in Fig. 1. Prior to initiating the device, samples are loaded into the stainless steel cylinder, followed by the insertion of metal perforated plate packing. Subsequently, the flotation liquid is prepared and the water pump, air pump, and suction filtration unit are activated. The flotation liquid is pumped into the bottom of the separation device, where air is introduced to thoroughly mix the air, flotation liquid, and samples. The mixture then passes through the metal perforated plate packing for separation and sedimentation. The microplastics are then directed towards the top conical overflow port and suctioned by the filtration unit into filters with different pore sizes, resulting in the separation of microplastics of varying particle sizes. Following digestion and drying treatment of the microplastics on the screen mesh and filter membrane, statistical analysis is conducted.

The addition of metal perforated plate packing (Fig. 1) to this design allows for the separation of microplastics and sediments. The microplastics with lower density float upwards, while the heavier sediments are intercepted and settle down within the packing under the force of gravity. The continuous infusion of air creates bubbles that, along with the flotation liquid, effectively flush the metal perforated plate packing, preventing the accumulation of sediments and microplastics. This ultimately accelerates the separation of microplastics and sediments. The overflow port is designed as a cone shape to facilitate the timely suction of separated microplastics into the filters. The filter unit consists of four independent, detachable, glass filters, allowing for convenient replacement in the field. The four filters are filled with a 1 mm stainless steel mesh, a 0.5 mm stainless steel mesh, a 0.1 mm stainless steel mesh, and a 1.6 μm glass fibre filter membrane.

## 3 Results and discussion

In order to identify the optimal parameters for this device, it is necessary to conduct parameter optimisation experiments. Recovery experiments were conducted using polystyrene (PS) particles with a diameter ranging from 0.5 to 1 mm and 0.1 to 0.5 mm, as well as polyethylene (PE) particles with a diameter ranging from 1 to 5 mm (all with 30 particles). The recovery rate was calculated by counting the recovered PS and PE particles and dividing them by the total number of particles initially used (90 particles), each group of experiments was run three times in parallel and the average of the results was taken.

### 3.1 Selection of metal perforated plate packing models

**Fig. 2:**
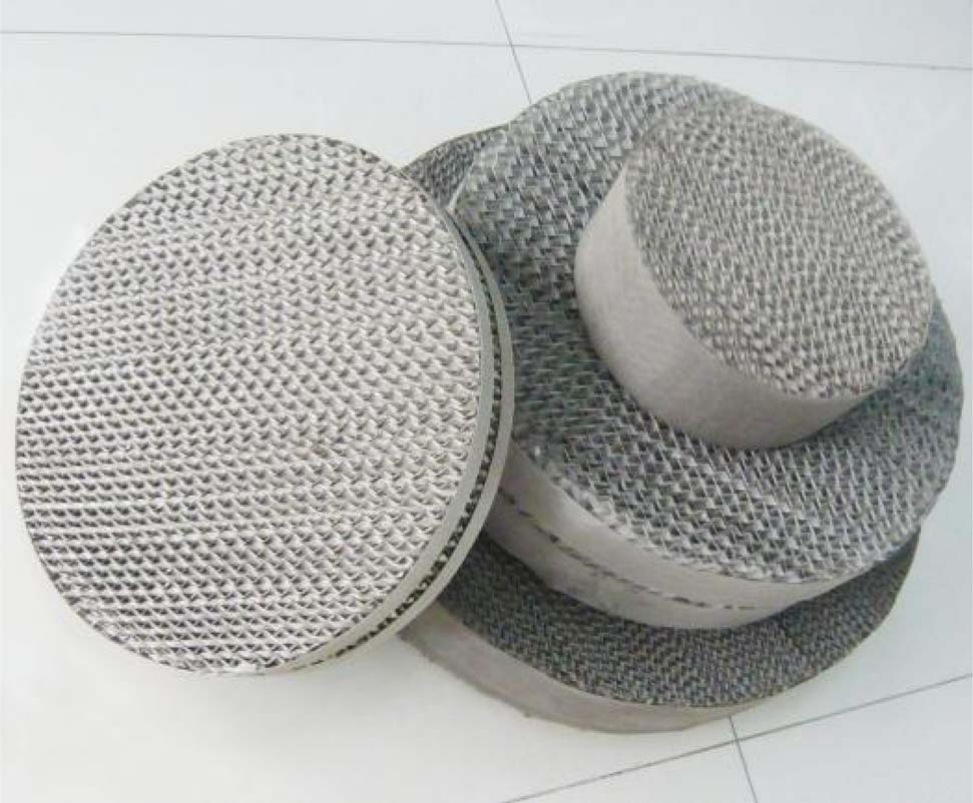
Metal perforated plate packing.

**Fig. 3:**
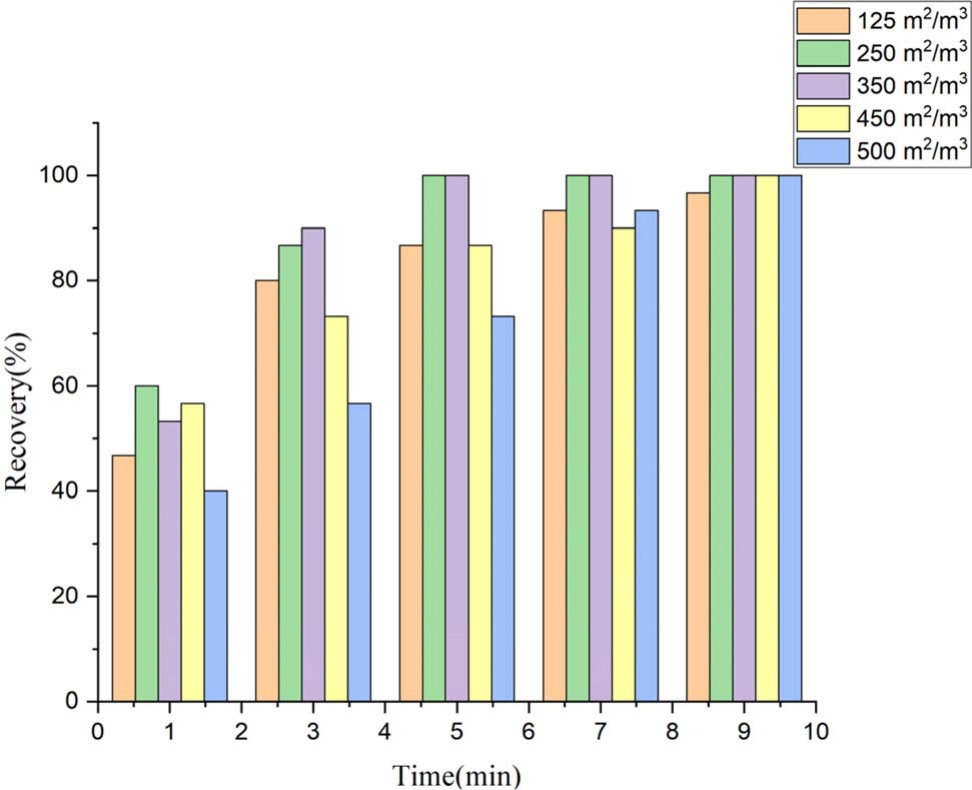
Effect of metal perforated plate packing on microplastic recovery rate.

In order to ascertain the impact of different types of metal perforated plate packing on the microplastic recovery rate, a saturated sodium iodide flotation liquid was used, with the gas flow set at 3 L min^−1^ and the flotation time set at 6 minutes. The images and parameters of the metal perforated plate packing are shown in Fig. 2 and Table 1, while their effect on the microplastic recovery rate is presented in Fig. 3.

**Table 1:** Parameters of metal perforated plate packing of different models

Model	Specific surface area (m^2^ m^−3^)	Void rate (%)	Peak height (Hmm)	Theoretical board count (Ntm)	Pressure drop (Mpa m^−1^)	Maximum *F*-factor (m s^−1^ (kg m^−2^)^−1^)
125	125	98	25.4	1–2	1.5 × 10^−4^	3.0
250	250	97	12.5	2–3	1.5–2 × 10^−4^	2.6
350	350	94	9.0	2.0–2.5	1.5–2 × 10^−4^	2.0
450	450	93	6.5	3–1	1.8 × 10^−4^	1.5
500	500	92	6.3	4–5	2 × 10^−4^	1.8

A comparison of the experimental results revealed that the metal perforated plate packing with specific surface areas of 250 m^2^ m^−3^ and 350 m^2^ m^−3^ could effectively complete the recovery of all microplastics within approximately five minutes. However, the metal perforated plate packing with specific surface areas of 125 m^2^ m^−3^, 450 m^2^ m^−3^, and 500 m^2^ m^−3^ required 7 to 9 minutes. Ultimately, the relatively inexpensive 250 m^2^ m^−3^ metal perforated plate filler was selected for the filling device in this study.

### 3.2 Selection of flotation solution

The most common flotation solutions currently in use include NaCl, NaI, ZnCl_2_, and others. NaCl is the most prevalent microplastic flotation salt solution due to its cost-effectiveness, non-toxicity, and ready availability. However, the density of a saturated NaCl solution is only 1.2 g cm^−3^, which enables the flotation of microplastics with lower densities such as PP and PE, but fails to float those with higher densities like PET, PVC, and POM, potentially underestimating pollution levels. In contrast, ZnCl_2_ has a saturated solution density ranging from 1.6 to 1.8 g cm^−3^, which is greater than the density of most plastics. Consequently, it is an effective microplastic flotation agent at a relatively low cost. However, its high toxicity and corrosive nature present significant risks during usage.^29^ NaI, with a saturated solution density similar to ZnCl_2_ at 1.8 g cm^−3^, offers excellent microplastic flotation performance without the toxicity and corrosiveness of ZnCl_2_. While its price is higher, it is considered a more cost-effective solution when the recyclability of the flotation solution in this device is taken into account. NaI saturated solution has been ultimately chosen as the flotation liquid, balancing both flotation efficiency and safety.

### 3.3 Selection of gas flow

**Fig. 4:**
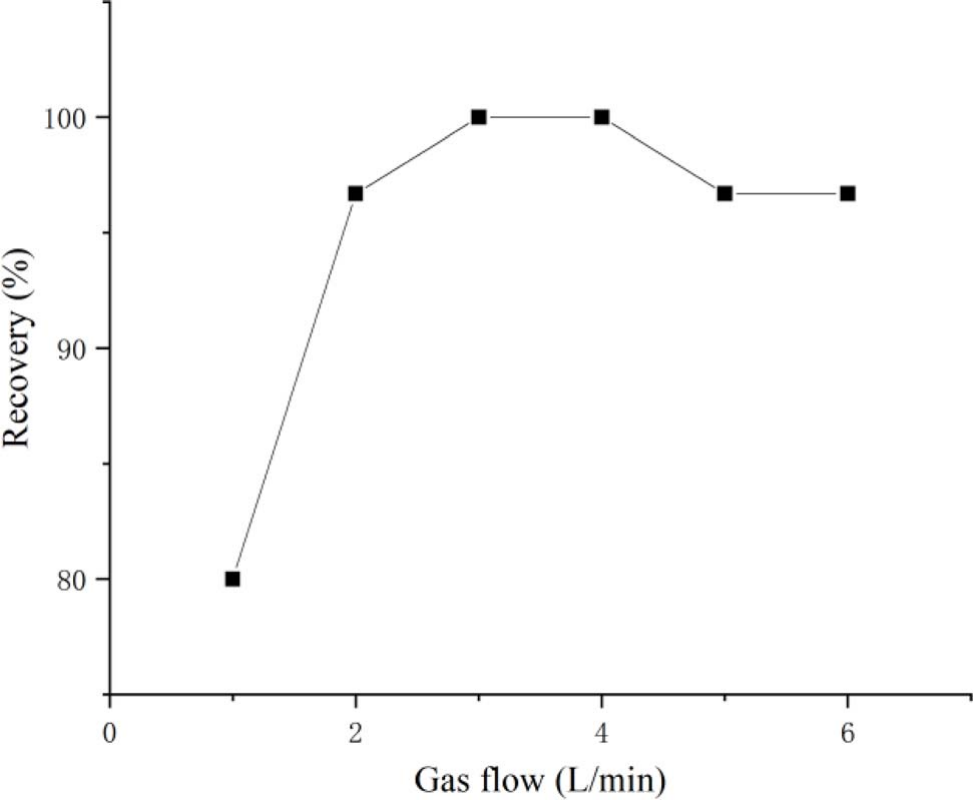
The effect of gas flow on the recovery of microplastics.

The flotation efficiency of microplastics is closely tied to the gas flow. Insufficient gas flow results in incomplete flotation of microplastics in the sample due to the lack of uniform mixing with the flotation liquid. Conversely, excessive gas flow can create large vortices in the device, extending the residence time of microplastics within the system and consequently reducing flotation efficiency. In order to ascertain the optimal gas flow for the recovery of microplastics, this study examined the impact of gas flow ranging from 1 to 6 L min^−1^. The results are depicted in Fig. 4. As seen in Fig. 4, the highest recovery were achieved at gas flow of 3 and 4 L min^−1^. Ultimately, this study opted for an gas flow of 3 L min^−1^.

### 3.4 Selection of flotation time

**Fig. 5:**
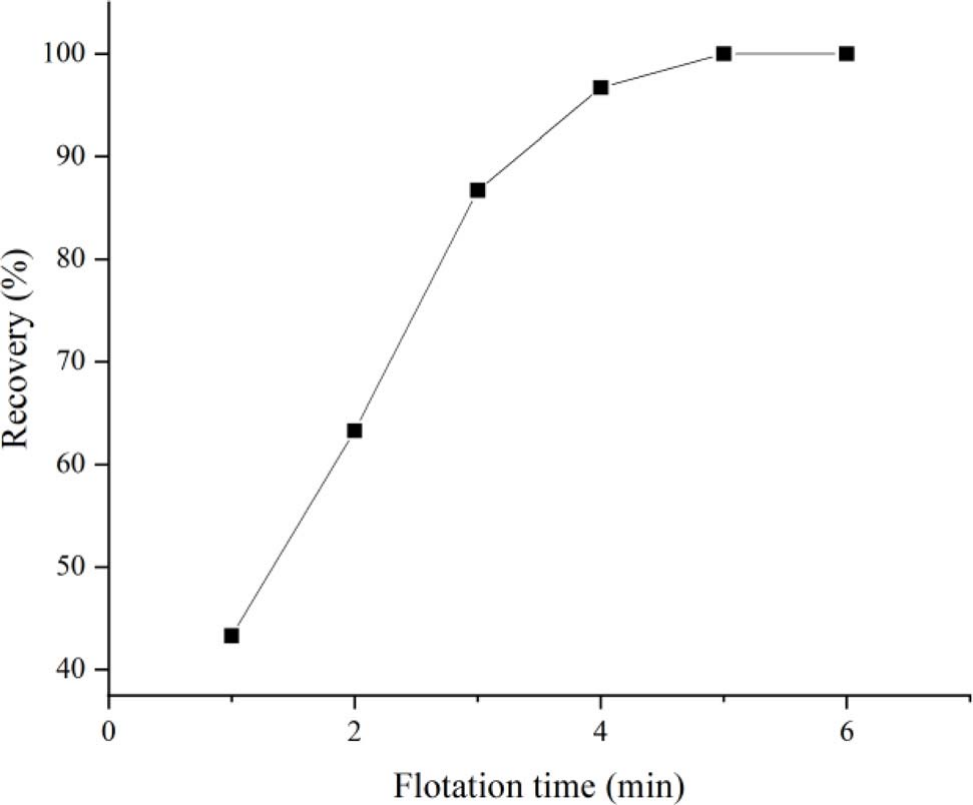
Effect of flotation time on microplastic recovery.

One of the most crucial factors influencing the efficiency of microplastic flotation is flotation time. In this study, flotation times ranging from 1 to 6 minutes were examined in order to ascertain the effect of flotation time on the recovery of microplastics. The results are presented in Fig. 5. As illustrated in Fig. 5, the recovery of microplastics gradually increases with longer flotation times. Upon reaching a flotation time of five minutes, the flotation effect of microplastics is deemed optimal. However, for added assurance, this study has set the flotation time at six minutes.

## 4 Application of microplastic extraction device

### 4.1 Sample

**Fig. 6:**
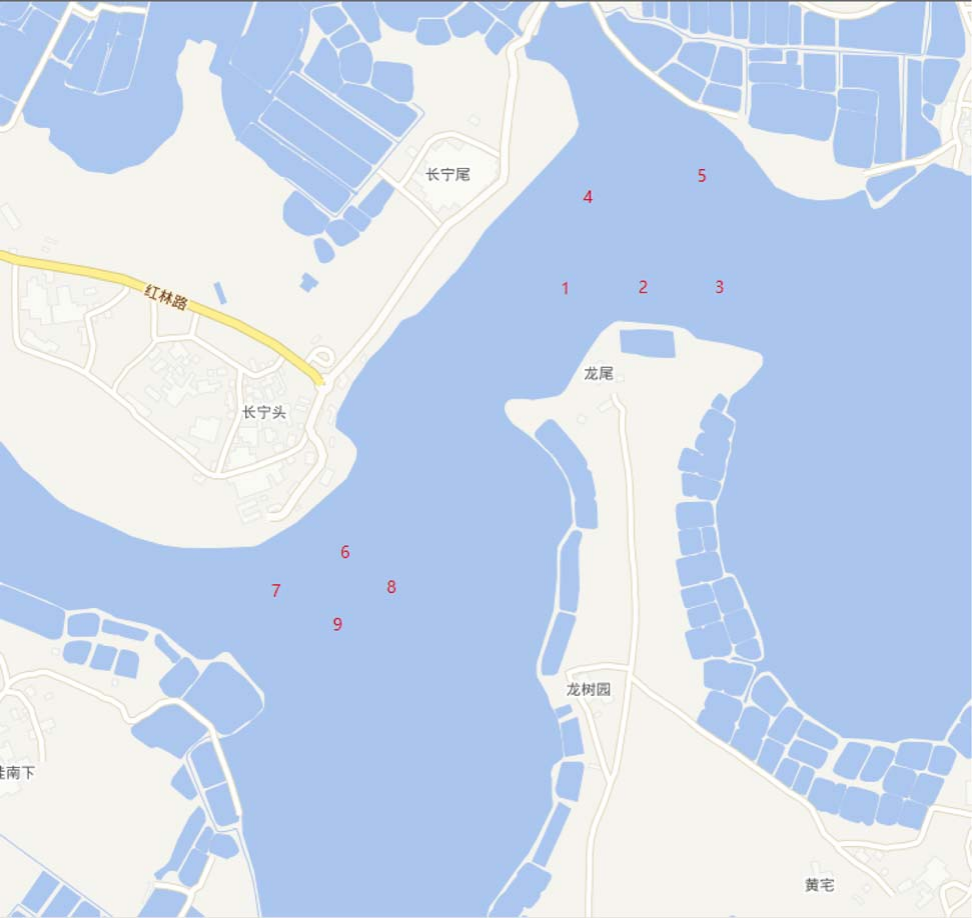
Sampling sites for microplastics.

In the mangrove area of Dongzhai Harbor, nine representative sampling sites were selected for the study. These sites were chosen to ensure that they were free from human disturbance. Sites 1 and 2 are located close to the sluice outlet, while Sites 3, 4, and 5 are situated near the inland area. Sites 6, 7, 8, and 9 are situated near the riverside dock. As illustrated in Fig. 6 and Table 2, at each sampling site, three quadrats of 50 cm × 50 cm × 2 cm were sequentially designated using a steel ruler, resulting in a total of 27 samples collected from the nine sampling sites. Subsequently, the samples were sealed in aluminium foil bags, labelled, and transported back to the laboratory for processing.

**Table 2:** Longitude and latitude of sampling sites

Site	Longitude and latitude
1	g110.58795286, 19.95184584
2	g110.58627944, 19.95188726
3	g110.58795286, 19.95184584
4	g110.58531415, 19.95358079
5	g110.58696605, 19.95368038
6	g110.58076648, 19.94751861
7	g110.57960832, 19.94679422
8	g110.58188175, 19.94689281
9	g110.58085221, 19.94620855

### 4.2 Sample processing and qualitative and quantitative analysis of microplastics

The collected samples were placed in an oven and dried at 60 °C for 48 hours. After remixing, 200 grams of samples were randomly weighed and sieved through steel sieves with pore sizes of 5 mm and 1 mm to separate larger gravel and plastics. Microplastics with particle sizes between 1–5 mm were preliminarily separated and identified visually. Subsequently, the sieved samples were placed in a microplastic extraction device for flotation extraction according to the set conditions. The microplastics that had been separated from the sieve mesh and filter membrane were rinsed into a 100 mL beaker using 30% H_2_O_2_. A quantity of 0.01 g of FeCl_2_ was added, and the beaker was sealed and heated in a water bath at a temperature of 60 °C for a period of 72 hours. This was done in order to remove biological organic matter from the samples. After a period of 24 hours, the samples were filtered with a glass fibre filter membrane (1.6 μm), dried, and then statistically classified for microplastics.

The dried filter membranes were placed under a stereomicroscope in order to observe their morphological and colour characteristics. The microplastics on the filter membranes were sorted, photographed using a microscope camera, and the sizes of the plastics were measured using a scale. The abundance, particle size, shape, colour, and other information of the microplastics were recorded separately. Due to the limitations of the sampling and processing methods used in this study, a 1.6 μm glass fibre filter membrane was used for filtration, so the lower limit of extractable microplastic particle size is 2 μm.

It is of paramount importance during the sample collection process to wear cotton clothes and gloves, and to use stainless steel spatulas and aluminium foil bags. Furthermore, it is of paramount importance to prevent the introduction of microplastics during the preprocessing and separation of samples, due to the potential for human error and the possibility of interference from dust in the air, which could affect the experimental results.

### 4.3 Results of microplastic content testing

#### 4.3.1 Quantity abundance

**Fig. 7:**
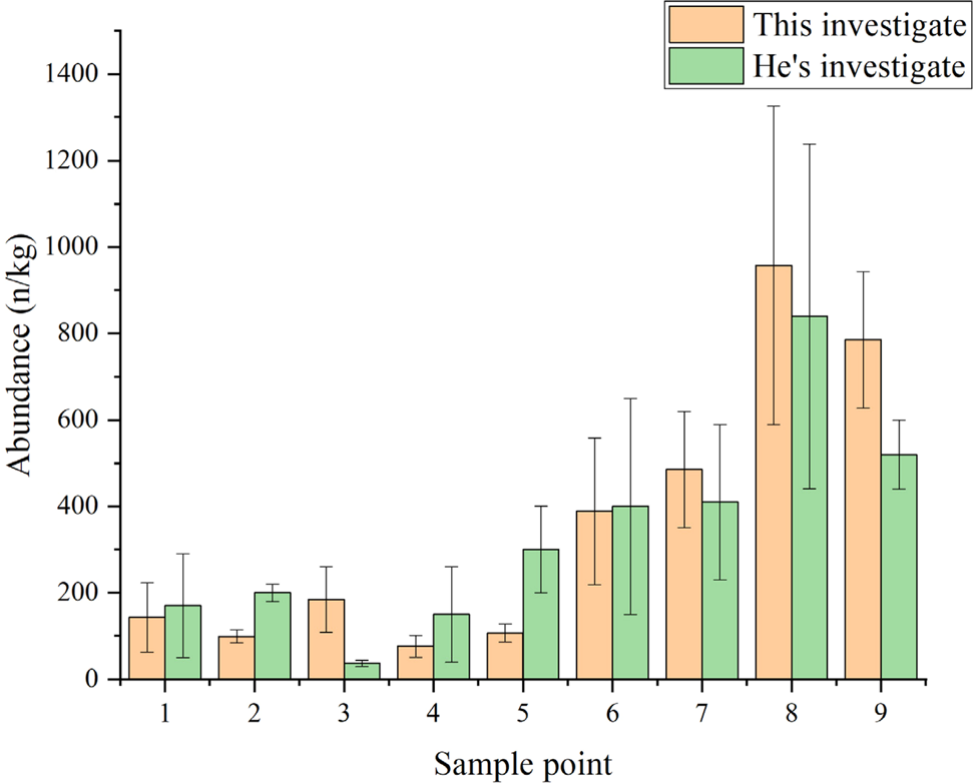
Quantity and abundance of microplastics at each sampling sites.

Each sampling point was detected with three replicates using a stereoscopic microscope and the results were expressed as means (±standard deviation). In addition, blank experiments were performed in this study and the results indicated that the background contamination during the experiment could be ignored. The results demonstrated the presence of microplastics at all sampling sites, with their quantitative abundance illustrated in Fig. 7. The number concentrations of microplastics ranged from 76.2 ± 25.2 to 957.6 ± 368.6 n kg^−1^ across 27 samples collected from the nine sampling sites, which aligns with the findings of He *et al.*^30^

#### 4.3.2 Particle size distribution

The particle size distribution of microplastics at different sampling sites was determined by analysing the microplastics collected on screens with various pore sizes and filter membranes. Table 3 presents the proportion of microplastics with different particle sizes at the nine sampling sites.

**Table 3:** Proportion of microplastic particle size at different sampling sites

Sample point	Particle size ratio		
	0.1–1 mm	1–2 mm	>2 mm
1	85.0	14.5	0.5
2	73.2	23.9	3.0
3	75.8	21.2	3.0
4	74.9	23.2	1.9
5	88.8	9.7	1.5
6	89.1	8.1	2.8
7	81.8	16.7	1.5
8	79.8	19.1	1.1
9	76.8	20.5	2.7

#### 4.3.3 Shape and composition distribution

Microplastics are classified into four types based on their shapes: fibrous, globular, fragment, and film. Table 4 outlines the percentages of these four shape categories of microplastics at the nine sampling sites. As can be seen from Table 4, fibrous microplastics account for the highest proportion in this region, primarily due to the high fishing activities in the area. The ageing and shedding of old fishing nets, ropes, and other materials can lead to contamination by fibrous microplastics. Fourier Infrared Spectroscopy results indicate that the fibrous are mainly composed of polypropylene and polyvinyl chloride; the film are polyethylene and polypropylene polymers; the globular are polystyrene; and the fragment are polyamide.

**Table 4:** Shape proportion of microplastics at different sampling sites

Sample point	Proportion (%)			
	Fibrous	Globular	Fragment	Film
1	17.9	27.0	17.0	38.1
2	49.3	23.2	24.0	3.5
3	45.8	24.3	2.3	27.6
4	57.3	28.9	12.5	1.3
5	52.5	17.4	26.6	3.6
6	44.8	18.0	7.4	29.8
7	65.0	12.7	7.0	15.3
8	50.1	10.3	4.9	34.7
9	40.5	17.5	4.9	37.1

## 5 Conclusion

(1) This paper presents an efficient extraction device for microplastics in sediments. By optimising the metal perforated plate fillers type, flotation liquid type, gasflow, and flotation time, the optimal extraction conditions for this device have been determined. Compared to the traditional density separation method, which often requires up to 24 hours of extraction time, the extraction time of this device is only a few minutes, significantly improving extraction efficiency. Furthermore, this device offers several advantages, including excellent extraction results and a simple operational design.

(2) To investigate the abundance, particle size distribution, shape, and composition of microplastics in the sediments of Dongzhai Harbor, Hainan, this device was utilised in conjunction with microscopy and Fourier Infrared Spectroscopy. The results were largely consistent with those reported in the literature, indicating the practicality of the microplastic extraction device designed in this paper.

## Data availability

The data supporting this article have been included as part of the ESI.†

## Conflicts of interest

The authors declare no conflict of interest.

## Supplementary Material

RA-014-D4RA04347B-s001
